# Astrocyte reactivity influences amyloid-β effects on tau pathology in preclinical Alzheimer’s disease

**DOI:** 10.1038/s41591-023-02380-x

**Published:** 2023-05-29

**Authors:** Bruna Bellaver, Guilherme Povala, Pamela C. L. Ferreira, João Pedro Ferrari-Souza, Douglas T. Leffa, Firoza Z. Lussier, Andréa L. Benedet, Nicholas J. Ashton, Gallen Triana-Baltzer, Hartmuth C. Kolb, Cécile Tissot, Joseph Therriault, Stijn Servaes, Jenna Stevenson, Nesrine Rahmouni, Oscar L. Lopez, Dana L. Tudorascu, Victor L. Villemagne, Milos D. Ikonomovic, Serge Gauthier, Eduardo R. Zimmer, Henrik Zetterberg, Kaj Blennow, Howard J. Aizenstein, William E. Klunk, Beth E. Snitz, Pauline Maki, Rebecca C. Thurston, Ann D. Cohen, Mary Ganguli, Thomas K. Karikari, Pedro Rosa-Neto, Tharick A. Pascoal

**Affiliations:** 1 Department of Psychiatry, University of Pittsburgh, Pittsburgh, PA USA; 2 Graduate Program in Biological Sciences—Biochemistry, Universidade Federal do Rio Grande do Sul, Porto Alegre, Brazil; 3 Department of Psychiatry and Neurochemistry, The Sahlgrenska Academy at the University of Gothenburg, Mölndal, Sweden; 4 Centre for Age-Related Medicine, Stavanger University Hospital, Stavanger, Norway; 5 Department of Old Age Psychiatry, Institute of Psychiatry, Psychology and Neuroscience, King’s College London, London, UK; 6 Neuroscience Biomarkers, Janssen Research and Development, La Jolla, CA USA; 7 Translational Neuroimaging Laboratory, McGill University Research Centre for Studies in Aging, Alzheimer’s Disease Research Unit, Douglas Research Institute, Le Centre intégré universitaire de santé et de services sociaux (CIUSSS) de l’Ouest-de-l’Île-de-Montréal; Department of Neurology and Neurosurgery, Psychiatry and Pharmacology and Therapeutics, McGill University, Montreal, Quebec Canada; 8 Department of Neurology, School of Medicine, University of Pittsburgh, Pittsburgh, PA USA; 9 Department of Biostatistics, School of Medicine, University of Pittsburgh, Pittsburgh, PA USA; 10 Geriatric Research Education and Clinical Center, VA Pittsburgh HS, Pittsburgh, PA USA; 11 Department of Pharmacology, Universidade Federal do Rio Grande do Sul, Porto Alegre, Brazil; 12 Graduate Program in Biological Sciences: Pharmacology and Therapeutics, Universidade Federal do Rio Grande do Sul, Porto Alegre, Brazil; 13 Brain Institute, PUCRS, Porto Alegre, Brazil; 14 Clinical Neurochemistry Laboratory, Sahlgrenska University Hospital, Mölndal, Sweden; 15 Department of Neurodegenerative Disease, UCL Queen Square Institute of Neurology, London, UK; 16 UK Dementia Research Institute at UCL, London, UK; 17 Hong Kong Center for Neurodegenerative Diseases, Hong Kong, China; 18 Wisconsin Alzheimer’s Disease Research Center, University of Wisconsin School of Medicine and Public Health, University of Wisconsin-Madison, Madison, WI USA; 19 Department of Bioengineering, University of Pittsburgh, Pittsburgh, PA USA; 20 Department of Psychiatry, University of Illinois, Chicago, IL USA; 21 Department of Psychology, University of Pittsburgh, Pittsburgh, PA USA; 22 Department of Epidemiology, University of Pittsburgh School of Public Health, Pittsburgh, PA USA; 23 Brain Imaging Centre, Montreal Neurological Institute-Hospital, Montreal, Quebec Canada

**Keywords:** Predictive markers, Predictive markers, Prognostic markers, Alzheimer's disease

## Abstract

An unresolved question for the understanding of Alzheimer’s disease (AD) pathophysiology is why a significant percentage of amyloid-β (Aβ)-positive cognitively unimpaired (CU) individuals do not develop detectable downstream tau pathology and, consequently, clinical deterioration. In vitro evidence suggests that reactive astrocytes unleash Aβ effects in pathological tau phosphorylation. Here, in a biomarker study across three cohorts (*n* = 1,016), we tested whether astrocyte reactivity modulates the association of Aβ with tau phosphorylation in CU individuals. We found that Aβ was associated with increased plasma phosphorylated tau only in individuals positive for astrocyte reactivity (Ast^+^). Cross-sectional and longitudinal tau–positron emission tomography analyses revealed an AD-like pattern of tau tangle accumulation as a function of Aβ only in CU Ast^+^ individuals. Our findings suggest astrocyte reactivity as an important upstream event linking Aβ with initial tau pathology, which may have implications for the biological definition of preclinical AD and for selecting CU individuals for clinical trials.

## Abstract (web summary)

Cross-sectional and longitudinal analyses of tau pathology in preclinical Alzheimer’s disease reveal that tau tangles accumulate as a function of amyloid-β burden only in individuals positive for an astrocyte reactivity biomarker.

## Main

Rapid advances in fluid and neuroimaging biomarkers have facilitated the understanding of the dynamic associations between Alzheimer’s disease (AD)-related pathophysiological processes in the living human brain. These biomarker studies suggest that brain accumulation of amyloid-β (Aβ) precedes tau pathology in cognitively unimpaired (CU) individuals^1–3^, which is closely related to the development of cognitive symptoms^4–6^. However, the reasons why Aβ pathology is not associated with AD-related progression in some CU individuals is one of the most pressing questions in the field^7–9^. In addition to revealing key biological players associated with disease progression, finding predictive markers of early Aβ-related tau pathology would allow for the identification of CU individuals who are more likely to develop AD even before the first signs of pathological tau, facilitating enrollment in early prevention clinical trials^10^.

The fact that Aβ leads to tau pathology in some individuals, but not in others, suggests the presence of other biological processes capable of triggering the deleterious effects of Aβ in the early disease stages. Postmortem studies show that astrocyte reactivity is a common neuropathological finding in CU individuals and, like cortical Aβ plaques, one of the earliest abnormalities in AD^11–15^. Experimental literature suggests that astrocyte reactivity is critical for triggering Aβ-induced tau phosphorylation^16^ and that the attenuation of astrocyte reactivity mitigates tau pathology^17,18^. Additionally, glial fibrillary acidic protein (GFAP)-positive astrocytes can internalize tau and may contribute to its propagation^19,20^. Altogether, these experimental results support a close link between Aβ, astrocyte reactivity and tau.

Clinical studies support that plasma measures of GFAP correlate with its CSF levels and are increased in CU individuals with AD pathophysiology, representing a robust proxy of astrocyte reactivity in the brains of these individuals^21–24^. Based on this previous literature, we designed a multisite biomarker study including three cohorts to test the hypothesis that the presence of astrocyte reactivity biomarker abnormality is a key element in determining the association of Aβ burden with early tau phosphorylation and aggregation biomarkers in preclinical AD.

## Results

### Participants

We investigated 1,016 CU individuals (mean age = 69.6 ± 8.9, clinical dementia rating (CDR) = 0) from two research (Translational Biomarkers in Aging and Dementia (TRIAD), McGill University, Canada and Pittsburgh, University of Pittsburgh, USA) and one population-based (Monongahela-Youghiogheny Healthy Aging Team (MYHAT), Pittsburgh, USA) cohorts with in vivo biomarkers. Individuals were classified as negative (Ast^−^) or positive (Ast^+^) for astrocyte reactivity based on their plasma GFAP levels. Demographic and clinical characteristics of participants are summarized in Table 1. Overall, participants classified as Aβ^+^/Ast^+^ presented increased plasma phosphorylated tau (p-tau)181, p-tau231 and p-tau217 compared to other groups. No differences in Aβ levels were observed between CU Aβ^+^/Ast^−^ and Aβ^+^/Ast^+^ in any cohort. Demographic characteristics of individuals segregated by cohort are presented in Supplementary Tables 1–3.

**Table 1:** Demographics and key characteristics of participants

Characteristics	Aβ^−^/Ast^−^ (*n* = 557)	Aβ^−^/Ast^+^ (*n* = 165)	Aβ^+^/Ast^−^ (*n* = 186)	Aβ^+^/Ast^+^ (*n* = 108)
Age, mean (s.d.)	68.2 (8.6)	72.1 (8.2)^a^	68.6 (7.6)^b^	74.7 (10.6)^a,c^
Sex, *n* (% female)	367 (65.9)	137 (83.0)^a^	122 (65.6)^b^	79 (73.1)
MMSE/MoCA, mean (s.d.)	28.1 (3.3)/27.5 (1.8)	28.1 (3.2)/28.1 (1.7)	27.8 (3.2)/26.7 (4.1)^b^	27.1 (6.1)/27.2 (1.4)
*APOE* ε4 (% of carriers)	89 (16.0)	25 (15.2)	33 (17.7)	25 (23.1)
Education, years (s.d.)	15.0 (2.7)	15.2 (3.1)	14.8 (2.7)	15.0 (2.8)
Aβ burden (*z* score, s.d.)	−0.52 (0.65)	−0.40 (0.57)	1.17 (0.63)^a,b^	1.25 (0.69)^a,b^
Plasma GFAP (*z* score, s.d.)	−0.50 (0.51)	1.20 (0.75)^a^	−0.42 (0.52)^b^	1.48 (0.88)^a,b,c^
Plasma p-tau181 (*z* score, s.d.)	−0.12 (0.90)	0.10 (0.82)^a^	−0.14 (0.97)	0.77 (1.89)^a,b,c^
Plasma p-tau231 (*z* score, s.d.)	−0.03 (1.00)	−0.12 (0.82)	−0.04 (0.86)	0.55 (1.26)^a,b,c^
Plasma p-tau217 (*z* score, s.d.)	−0.27 (0.38)	−0.24 (0.38)	0.17 (0.49)	1.12 (1.99)^a,b,c^
Plasma neurofilament light (*z* score, s.d.)	−0.24 (0.62)	0.42 (0.99)^a^	−0.13 (1.22)^b^	0.80 (1.47)^a,b,c^

Missing *APOE* ε4: 140 Aβ^−^/Ast^−^, 43 A^−^/Ast^+^, 45 Aβ^+^/Ast^−^ and 17 Aβ^+^/Ast^+^. Missing neurofilament light: 2 Aβ^−^/Ast^−^, 2 Aβ^−^/Ast^+^ and 1 Aβ^+^/Ast^−^. Plasma p-tau231 is available for a subset of participants from TRIAD and Pittsburgh cohorts. Plasma p-tau217 is available for a subset of participants from the TRIAD cohort.

^a^Different from Aβ^−^/Ast^−^.

^b^Different from Aβ^−^/Ast^+^.

^c^Different from Aβ^+^/Ast^−^.

### Astrocyte reactivity affects Aβ-dependent tau phosphorylation

**Fig. 1:**
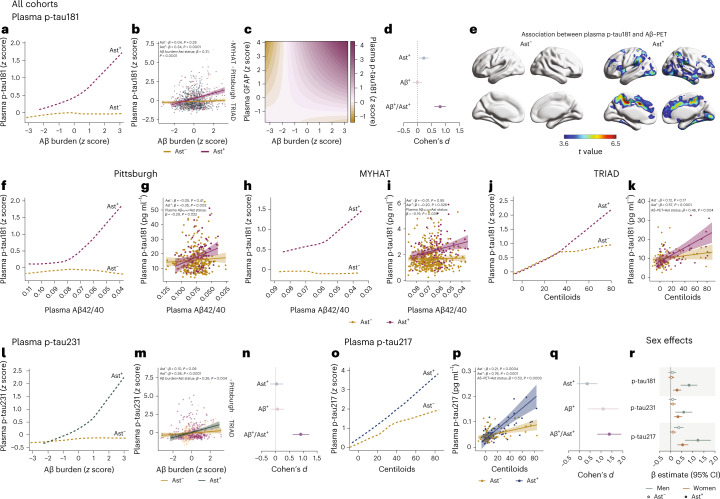
Astrocyte reactivity influences Aβ-dependent tau phosphorylation. **a**, Robust linear regressions show that plasma p-tau181 increases as a function of Aβ burden only in the presence of astrocyte reactivity (Ast^+^) in all cohorts together (*n* = 1,016). **b**, Linear regressions adjusted for age and sex revealed an interaction between Aβ burden and astrocyte reactivity status on p-tau181 levels in all cohorts (*n* = 1,016). Shaded areas represent 95% confidence intervals of the regression lines. **c**, Continuous association between Aβ pathology, plasma p-tau181 and plasma GFAP adjusted for age and sex (*n* = 1,016). **d**, Cohen’s *d* analysis accounting for age and sex shows the effect sizes of Aβ and astrocyte reactivity status on plasma p-tau181 (*n* = 1,016). The error bars represent the 95% confidence interval. **e**, Voxel-wise regressions, corrected for multiple comparisons, show that Aβ–PET is associated with plasma p-tau181 only in CU Ast^+^ in typical AD regions (TRIAD cohort, *n* = 147). **f**–**k**, Robust locally weighted and linear regressions adjusted for age and sex show that plasma p-tau181 increases as a function of Aβ burden only in Ast^+^ individuals and with a significant interaction between Aβ and astrocyte reactivity status on p-tau181 levels in (**f**,**g**) Pittsburgh (*n* = 355), (**h**,**i**) MYHAT (*n* = 514) and (**j**,**k**) TRIAD (*n* = 147) cohorts. Shaded areas represent 95% confidence intervals of the regression lines. **l**,**m**, Robust locally weighted and linear regressions adjusted for age and sex show that (**l**) plasma p-tau231 increases as a function of Aβ burden only in Ast+ individuals and with (**m**) a significant interaction between Aβ burden and astrocyte reactivity status on p-tau231 (*n* = 502). **n**, Cohen’s *d* analysis accounting for age and sex shows the effect sizes of Aβ and astrocyte reactivity status on plasma p-tau231 (*n* = 502). The error bars represent the 95% confidence intervals. **o**,**p**, Robust locally weighted and linear regressions adjusted for age and sex show that (**o**) plasma p-tau217 increases as a function of Aβ burden only in Ast+ individuals and with (**p**) a significant interaction between Aβ burden and astrocyte reactivity status on p-tau217 (*n* = 136). Shaded areas represent 95% confidence intervals of the regression lines. For illustrative purposes only, two individuals with high plasma p-tau181 and p-tau217 concentrations were not shown in **k** and **p**, but they were fully included in the statistical analyses. **q**, Cohen’s *d* analysis accounting for age and sex shows the effect sizes of Aβ and astrocyte reactivity status on plasma p-tau217 (*n* = 136). The error bars represent the 95% confidence intervals. **r**, β estimates with respective 95% confidence interval of linear regressions showing the effect of sex on the associations of Aβ with plasma p-tau epitopes in Ast^−^ and Ast^+^ (*n* = 1,016). Green dots represent men and orange dots women. Solid dots represent Ast^+^ individuals.

We *z* scored biomarker levels inside each cohort and applied a robust linear regression to model the trajectory of plasma p-tau181, the only p-tau biomarker available in all cohorts, as a function of Aβ burden (plasma or positron emission tomography (PET)) in CU individuals classified as Ast^−^ (*n* = 743) or Ast^+^ (*n* = 273). Notably, we observed that plasma p-tau181 levels increased as a function of Aβ only in CU Ast^+^ individuals (Fig. 1a). Similarly, linear regression showed a significant association between Aβ burden and plasma p-tau181 in CU Ast^+^ (*β* = 0.34, *t* = 5.37, *P* < 0.0001; Fig. 1b and Extended Data Table 1) but not in CU Ast^−^ (*β* = 0.04, *t* = 1.06, *P* = 0.29; Fig. 1b) individuals. A significant interaction between Aβ burden and astrocyte reactivity status on plasma p-tau181 (*β* = 0.31, *t* = 4.62, *P* < 0.0001; Fig. 1b) further supported that the presence of astrocyte reactivity was key to determining Aβ effects on tau phosphorylation. These results were replicated using different cutoff values to determine reactive astrocyte positivity (Extended Data Table 2). In addition, linear regression using only continuous values for Aβ, p-tau181 and GFAP levels confirmed that these results were not driven by biomarker thresholds (*β* = 0.10, *t* = 3.22, *P* = 0.0013; Fig. 1c). Cohen’s *d* analysis revealed that the presence of both Aβ^+^ and Ast^+^ has a large magnitude of effect on tau phosphorylation (Cohen’s *d* = 0.80), whereas Aβ^+^ in the absence of Ast^+^ presented a negligible effect size (Fig. 1d). Voxel-wise analysis confirmed that Aβ levels associated with plasma p-tau181 only in the presence of astrocyte reactivity in some brain regions previously shown to present early Aβ pathology in PET studies, including the posterior cingulate, precuneus and insula^25^ (Fig. 1e).

**Extended Data Fig. 1:**
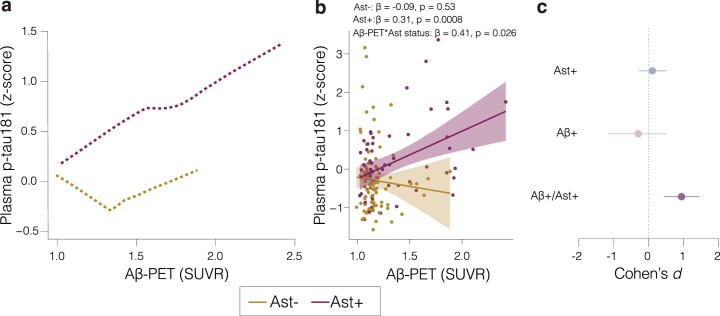
Impact of astrocyte reactivity in the association between Aβ-PET and plasma p-tau181 in a subset of individuals from MYHAT and Pittsburgh cohorts. **a**, Robust locally weighted regressions show that plasma p-tau181 increases as a function of Aβ-PET only in Ast+ (*n* = 150). **b**, Linear regressions revealed an interaction between Aβ-PET and astrocyte reactivity status on p-tau181 levels (*n* = 150). The plots show regression line with their respective 95% confidence intervals. P-values were computed using linear regression models adjusted by age and sex. In addition, the Aβ-PET × astrocyte reactivity status interaction was also computed. Red lines and dots represent Ast+ and orange lines and dots represent Ast− individuals. **c**, Cohen’s *d* analysis accounting for age and sex shows the effect sizes of Aβ and Ast on plasma p-tau181 (*n* = 150). The error bars denote the 95% confidence intervals.

Consistently, the stratified analysis within cohorts showed similar results. In the three enrollment sites, plasma p-tau181 levels increased as a function of Aβ burden only in CU Ast^+^ (Pittsburgh: *β* = −0.35, *t* = 3.10, *P* = 0.003 (Fig. 1f); MYHAT: *β* = −0.20, *t* = 2.26, *P* = 0.026 (Fig. 1h) and TRIAD: *β* = 0.57, *t* = 4.36, *P* < 0.0001 (Fig. 1j)). A steeper increase in plasma p-tau181 was observed in the research cohorts (TRIAD and Pittsburgh) compared to the population-based cohort (MYHAT). Similarly, we observed a significant interaction between Aβ burden and astrocyte reactivity status on plasma p-tau181 levels in the Pittsburgh (*β* = −0.29, *t* = 2.30, *P* = 0.022; Fig. 1g), MYHAT (*β* = −0.19, *t* = 2.07, *P* = 0.039; Fig. 1i) and TRIAD (*β* = 0.46, *t* = 2.92, *P* = 0.004; Fig. 1k) cohorts. In a subset of participants from the Pittsburgh and MYHAT cohorts with available Aβ–PET (*n* = 150), we also found increased plasma p-tau181 as a function of Aβ–PET only in Ast^+^ (Extended Data Fig. 1). No significant effect of apolipoprotein ε4 (*APOE ε4*) status was observed in the aforementioned associations (Extended Data Table 3).

We also explored the impact of Ast^+^ in the associations of Aβ burden with plasma p-tau231 (available for Pittsburgh and TRIAD cohorts, *n* = 502) and p-tau217 (available for the TRIAD cohort, *n* = 136) levels in subsets of individuals that had these markers available. Plasma p-tau231 increased as a function of Aβ only in CU Ast^+^ individuals (Fig. 1l). Additionally, we found a significant association between Aβ and plasma p-tau231 in CU Ast^+^ (*β* = 0.36, *t* = 4.62, *P* < 0.0001; Fig. 1m and Extended Data Table 3) but not in CU Ast^−^ individuals (*β* = 0.10, *t* = 1.87, *P* = 0.06). We also observed a significant interaction between Aβ and astrocyte reactivity status on plasma p-tau231 (*β* = 0.26, *t* = 2.84, *P* = 0.004; Fig. 1m). Cohen’s *d* analysis suggests that the presence of both Aβ^+^ and Ast^+^ also had a strong effect on the levels of plasma p-tau231 (Cohen’s *d* = 0.91), whereas pathologies independently did not have a significant effect (Fig. 1n). Similarly, plasma p-tau217 presented a steeper increase as a function of Aβ burden in Ast^+^ compared to Ast^−^ (Fig. 1o and Extended Data Table 4). An association between Aβ burden and plasma p-tau217 was observed in Ast^−^ (*β* = 0.21, *t* = 3.74, *P* = 0.0004; Fig. 1p), but with a much larger magnitude in Ast^+^ (*β* = 0.76, *t* = 5.91, *P* < 0.0001; Fig. 1p). The stronger association in CU Ast^+^ individuals was further evidenced by a significant interaction between Aβ burden and astrocyte reactivity status on plasma p-tau217 (*β* = 0.53, *t* = 3.74, *P* = 0.0003; Fig. 1p). The presence of both Aβ^+^ and Ast^+^ had the largest effect size on plasma p-tau217 increase (Cohen’s *d* = 1.41; Fig. 1q) compared to p-tau181 and p-tau231. Notably, the presence of astrocyte reactivity did not impact the association between Aβ burden and neurofilament light levels in any of the three cohorts (Supplementary Table 4), supporting that astrocyte reactivity unleashes Aβ effects on early tau pathology but not on neurodegeneration. We did not observe a significant effect of microglial activation abnormality on the association between Aβ and p-tau in a subset of individuals with available CSF soluble triggering receptor expressed on myeloid cells 2 (sTREM2) in the TRIAD cohort, whereas the effect of astrocyte reactivity remained present in this subgroup (*n* = 67; Extended Data Table 5).

### Sex affects the association of astrocyte reactivity, Aβ and tau

**Extended Data Fig. 2:**
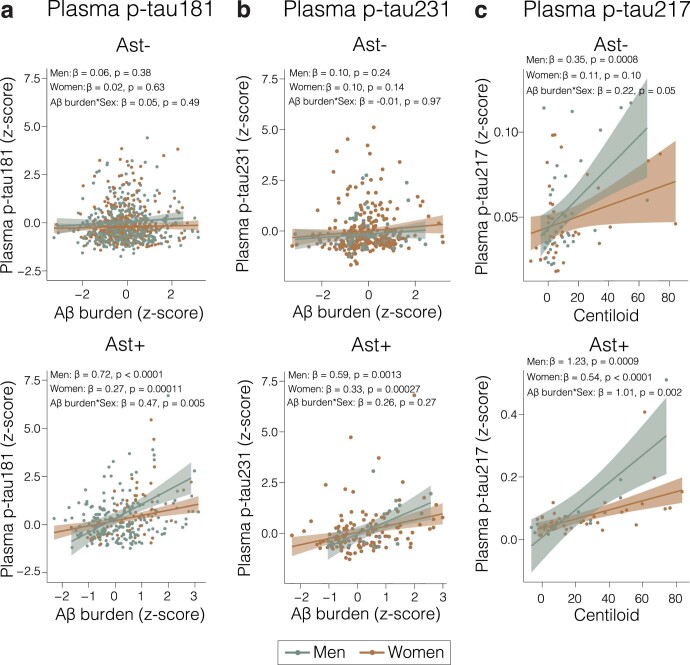
The impact of astrocyte reactivity on the Aβ and tau association is greater in men. Linear regressions showing the effect of sex on the associations of Aβ with (**a**) plasma p-tau181, (**b**) plasma p-tau231 and (**c**) plasma p-tau217 in individuals negative (Ast−, top) and positive (Ast+, bottom) for astrocyte reactivity. The plots show regression line with their respective 95% confidence intervals and the p-values were adjusted by age and sex. In addition, a plasma Aβ burden × sex interaction was also computed. Orange lines and dots represent women and green lines and dots represent men.

We further investigated whether the effects of astrocyte reactivity on the association between Aβ and p-tau differ between men and women. In Ast^+^, men presented a higher magnitude of association between Aβ and plasma p-tau181 (men—*β* = 0.72, *P* < 0.0001; women—*β* = 0.27, *P* = 0.00011; Fig. 1r; Extended Data Fig. 2 and Supplementary Fig. 1), p-tau231 (men—*β* = 0.59, *P* = 0.0013; women—*β* = 0.33, *P* = 0.0003; Fig. 1r and Extended Data Fig. 2) and p-tau217 (men—*β* = 1.23, *P* = 0.0009; women—*β* = 0.54, *P* < 0.0001; Fig. 1r and Extended Data Fig. 2) compared to women. A significant interaction between Aβ and sex on plasma p-tau181 (*β* = 0.47, *P* = 0.005; Extended Data Fig. 2) and p-tau217 (*β* = 1.01, *P* = 0.002; Extended Data Fig. 2), but not p-tau231 (*β* = 0.26, *P* = 0.27; Extended Data Fig. 2), was observed in the Ast^+^ group.

### Astrocyte reactivity impacts Aβ and tau tangle association

**Fig. 2:**
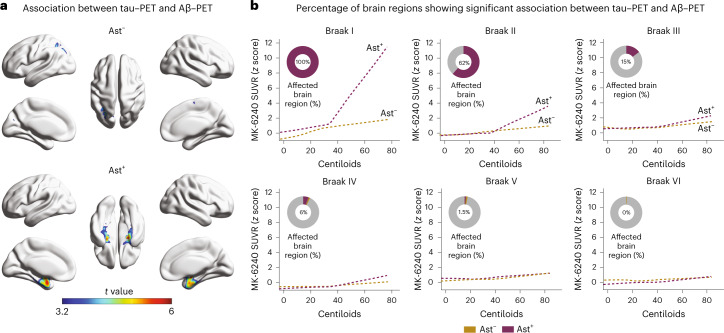
Astrocyte reactivity impacts the association of Aβ with tau–PET deposition. **a**, Voxel-wise regression analysis showing the association between Aβ–PET and tau–PET in individuals classified as negative (Ast^−^) or positive (Ast^+^) for astrocyte reactivity (*n* = 147). **b**, Percentage of the extent of the brain region with significant association (after RFT correction) between tau–PET and Aβ–PET in each Braak region. Associations were tested using voxel-wise linear regression models corrected for RFT multiple comparison and adjusted by age and sex. RFT, random field theory.

We used tau–PET imaging available in the TRIAD cohort to determine the topographic localization of p-tau protein aggregates in the form of tangles (*n* = 147). Tau–PET deposition occurred as a function of Aβ–PET only in CU Ast^+^ and in regions expected to present the earliest tau deposition (Fig. 2a), affecting 100% and 62% of the extension of the Braak I and II regions, respectively (Fig. 2b). As expected, in later Braak regions tau–PET, uptake did not increase as a function of Aβ in either group (Fig. 2b).

### Astrocyte reactivity affects longitudinal tau tangle accumulation

**Fig. 3:**
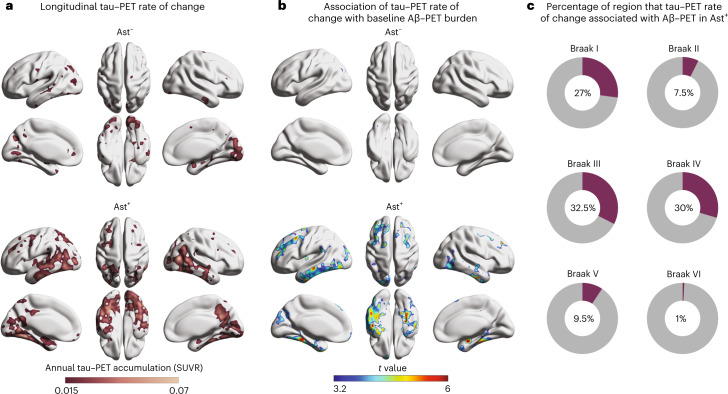
Astrocyte reactivity potentiates longitudinal tau tangle accumulation. **a**, Longitudinal tau–PET annual rate of change according to astrocyte reactivity status (*n* = 71). **b**, Association between tau–PET annual rate of change and baseline Aβ–PET according to astrocyte reactivity status. **c**, Percentage of voxels with significant association (after RFT correction) between tau–PET annual rate of change and baseline Aβ–PET in each Braak region. Associations were tested using voxel-wise linear regression models corrected for RFT multiple comparison and adjusted by age and sex.

We investigated the link of baseline Aβ and astrocyte reactivity status with future tau–PET burden (*n* = 71; mean follow-up = 2.3 years; Supplementary Table 5). We observed that the annual rate of tau–PET accumulation was higher in CU Ast^+^ (Fig. 3a) and was predicted by baseline Aβ burden only in CU Ast^+^ (Fig. 3b). Interestingly, while the baseline association was confined to the mesial temporal cortex, the longitudinal tau–PET accumulation as a function of Aβ/Ast presented initial tau spread over the neocortex in Braak III–IV regions (32.5% of Braak III and 30% of Braak IV areas; Fig. 3c), further supporting the notion that these individuals are following a tau accumulation pathway consistent with AD progression^26^.

## Discussion

In summary, we provide biomarker evidence across multiple cohorts that shows that increased astrocyte reactivity, as indicated by elevated plasma GFAP, plays a role in the association of Aβ with early tau phosphorylation in preclinical AD.

The fact that the presence of abnormal astrocyte reactivity determined Aβ-triggered tau pathology in CU individuals may prove to favor the inclusion of astrocyte reactivity biomarkers in the biomarker modeling^1^ and biological definitions^27^ of AD. Our findings support previous biomarker studies in suggesting that plasma p-tau levels rise in response to early Aβ in preclinical AD^3^ but also add that this occurs mainly in the concomitant presence of astrocyte reactivity biomarker abnormality. These results suggest that astrocyte reactivity abnormality could be placed as an early upstream event, likely before tau pathology, in the hypothetical biomarker models of AD progression^28^. Furthermore, we can argue that an Aβ/Ast/p-tau biomarker scheme could provide a more granular early classification for preclinical AD. Specifically, CU classified as Aβ^+^ and Ast^+^ would be more likely to progress to tau positivity, which is closely related to the development of neurodegeneration and cognitive decline^6,29,30^. Further studies measuring Aβ, tau and GFAP biomarkers at multiple time points with long follow-up durations are needed to confirm the temporal order of appearance of each biomarker abnormality and to determine other possible factors associated with early astrocyte reactivity in preclinical AD.

Our results may have implications for clinical trials. As clinical trials have increasingly focused on individuals in the earliest preclinical phases of AD, our results highlight that the selection of Aβ^+^/Ast^+^ CU individuals without overt p-tau abnormality may offer a time window very early in the disease process but with an increased risk of AD-related progression. Although our sensitivity analysis supported plasma GFAP levels 2 s.d. above the mean of CU devoid of detectable Aβ as a robust cutoff to enhance Aβ and p-tau association, a cutoff validation using antemortem blood samples associated with postmortem GFAP characterization is desirable. In addition, experimental literature suggests as a possible mechanism underlying our findings that Aβ-mediated astrocyte signaling (for example, through cytokines, caspases and reactive oxygen species) induces tau pathology^3,13,29^, which is corroborated by studies showing that the suppression of this signaling is able to halt tau phosphorylation^17^. Thus, we can speculate that a combination of drugs targeting Aβ and reactive astrocyte mediators may potentiate the prevention of early tau pathology in trials conducted in preclinical AD individuals.

We found that the effect of astrocyte reactivity on the association between Aβ and tau phosphorylation was greater in CU men than women. Previous studies have shown increased tau biomarkers in women compared to men; however, whether this is driven by Aβ or other sex-specific factors remains unclear^31–33^. Therefore, our results showing that astrocyte reactivity had a greater impact on the association of Aβ with tau in men does not contradict the above-mentioned previous studies. In fact, our findings might be reflected in the recent outcomes of anti-Aβ therapies, which might be modifying this Aβ–astrocyte–tau pathway, showing a higher magnitude of effect in men than women^34^.

The strengths of our study include a large sample size for the main analysis and the use of well-characterized research and population-based cohorts. Limitations include the fact that the analysis using longitudinal tau–PET had a relatively small sample size; thus, replication of this finding is highly desirable. As biomarkers are naturally continuous, dichotomizing cutoffs are invariably subjected to conceptual and analytical idiosyncrasies and may change depending on the method used. Finally, although our cohort represents significant socioeconomic diversity, the main limitation is that our cohorts are composed mainly of White participants, which limits the generalizability of our findings to a more diverse world population.

In conclusion, our findings suggest that detecting astrocyte reactivity biomarker abnormality is critical to predict whether CU Aβ-positive individuals will develop tau pathology and, consequently, clinical symptoms.

## Methods

### Study population

This study included participants from three centers. The TRIAD cohort (Montreal, Canada, https://triad.tnl-mcgill.com) comprised participants with a detailed clinical and cognitive assessment. Exclusion criteria included inability to speak English or French; inadequate visual and auditory capacities for neuropsychological assessment; active substance abuse; major surgery; recent head trauma; medical contraindication for PET or magnetic resonance imaging; currently being enrolled in other studies and neurological, psychiatric or systemic comorbidities that were not adequately treated with a stable medication regimen. CU individuals had a CDR = 0 and no objective cognitive impairment. The study was approved by the Douglas Mental Health University Institute Research Ethics Board and Montreal Neurological Institute PET working committee, and all participants provided written informed consent.

The MYHAT is a population-based study cohort drawn from a Rust Belt region of southwestern Pennsylvania, USA^35^. Participants were selected by age-stratified random sampling from the publicly available voter registration lists over the following two time periods: 2006–2008 and 2016–2019. Inclusion criteria at study entry included the following: (1) 65+ years old, (2) living in a designated town, (3) not residing in long-term care settings, (4) having sufficient hearing and vision to complete neuropsychological testing and (5) having decisional capacity. CU individuals had CDR = 0. All study procedures were approved by the University of Pittsburgh Institutional Review Board, and all participants provided written informed consent.

The Pittsburgh cohort is composed of research volunteers from the following four studies conducted at the University of Pittsburgh: the Heart Strategies Concentrating on Risk Evaluation parent study^36^, the Human Connectome Project^37^, the Normal Aging study^38^ and the MsBrain study^39^. All individuals provided written informed consent to one of the above studies. CU individuals were classified using either CDR = 0 or Montreal Cognitive Assessment (MoCA) >25 (for the MsBrain study). Individuals were selected according to cognitive status and plasma biomarker availability. Details of each cohort recruitment are reported in Supplementary Table 6. All study procedures were approved by the University of Pittsburgh Institutional Review Board, and all participants provided written informed consent.

Participants from all cohorts received compensation commensurate with the number of study procedures completed and the duration of participation.

### Plasma and CSF biomarkers

For Pittsburgh and TRIAD cohorts, plasma biomarkers (except for plasma p-tau217) were measured using single-molecule array (Simoa) methods on an HD-X Automated Immunoassay Analyzer (Quanterix), at the Clinical Neurochemistry Laboratory at the University of Gothenburg. Plasma Aβ42, Aβ40, GFAP and neurofilament light were quantified with the Neurology 4-Plex E (103670) commercial assays from Quanterix. Plasma p-tau181 (ref. ^40^) and plasma p-tau231 (ref. ^41^) were measured using an in-house Simoa assay developed at the University of Gothenburg, as previously described. Plasma p-tau217 (available only for TRIAD) was quantified by scientists at Janssen Research & Development^42^. For the MYHAT cohort, plasma biomarkers were measured using Simoa methods on an HD-X instrument (Quanterix), at the Department of Psychiatry, University of Pittsburgh School of Medicine. Plasma p-tau181 was measured with the p-tau181 V2 Advantage (103714), whereas plasma Aβ42, Aβ40, GFAP and neurofilament light concentrations were measured with the Neurology 4-Plex E (103670) commercial assays from Quanterix. For cohorts with no available Aβ–PET (that is, MYHAT and Pittsburgh cohorts), Aβ positivity was determined using plasma Aβ42/40 based on the expected 30% of Aβ positivity in CU individuals^43^. As younger individuals are more likely to be devoid of AD-related pathology^44,45^, cutoffs for astrocyte reactivity were generated using the plasma GFAP concentration mean of the 15% youngest Aβ-negative individuals plus 2 s.d. Sensitivity analyses using multiple cutoffs to define Aβ positivity or astrocyte positivity can be found in Supplementary Fig. 2 and Extended Data Table 2. CSF sTREM2 was measured using a Meso-Scale Discovery assay in a subsample in the TRIAD cohort, and the cutoff for positivity was set to correspond to the same percentile of GFAP positivity^46^.

### Magnetic resonance imaging/PET biomarkers

For the TRIAD cohort, Aβ–PET was quantified using the tracer (^18^F)AZD4694 and tau–PET with the tracer (^18^F)MK-6240 in a Siemens high-resolution research tomograph. (^18^F)AZD4694 and (^18^F)MK-6240 images were acquired at 40–70 min and 90–110 min postinjection, respectively. Standardized uptake value ratio (SUVR) was calculated using the whole cerebellum gray matter for (^18^F)AZD4694 and inferior cerebellum gray matter (^18^F)MK-6240 as the reference tissue. Neocortical (^18^F)AZD4694 SUVR value was estimated from a cortical composite, including the precuneus, prefrontal, orbitofrontal, parietal, temporal, anterior and posterior cingulate cortices. Individuals with Aβ–PET neocortical SUVR > 1.55 were considered Aβ-positive^47^. A subsample of 71 CU individuals had a follow-up (^18^F)MK-6240 with a mean of 2.3 years after baseline. Tau–PET Braak stage segmentation has been previously described^48^. Stages consisted of the following regions: Braak I (transentorhinal), Braak II (entorhinal and hippocampus), Braak III (amygdala, parahippocampal gyrus, fusiform gyrus and lingual gyrus), Braak IV (insula, inferior temporal, lateral temporal, posterior cingulate and inferior parietal), Braak V (orbitofrontal, superior temporal, inferior frontal, cuneus, anterior cingulate, supramarginal gyrus, lateral occipital, precuneus, superior parietal, superior frontal and rostromedial frontal) and Braak VI (paracentral, postcentral, precentral and pericalcarine).

A subset of individuals from the MYHAT (*n* = 86) and Pittsburgh (*n* = 64) cohorts had Aβ–PET available for this study. For these individuals, Aβ–PET was quantified using (^11^C)PiB PET acquired at 50–70 min postinjection. A global (^11^C)PiB SUVR index was computed by volume-weighted averaging of all nine composite (^11^C)PiB regions (anterior cingulate, posterior cingulate, insula, superior frontal cortex, orbitofrontal cortex, lateral temporal cortex, parietal, precuneus and ventral striatum). Aβ–PET positivity was defined using a previously established cutoff^49^.

### Statistical analysis

Neuroimaging analyses were carried out using the VoxelStats toolbox version 1 (https://github.com/sulantha2006/VoxelStats), a MATLAB‐based analytical framework that allows for the execution of multimodal voxel‐wise neuroimaging analyses^50^ and an R Package for Medical Imaging NetCD (RMINC) v1.5.3.0. Other statistical analyses were performed using the R Statistical Software Package version 3.5.3. Differences between groups in continuous variables (age, cognitive performance (mini-mental state exam (MMSE) or MoCA), biomarkers for Aβ, plasma GFAP, p-tau epitopes and neurofilament light) were assessed using analysis of variance with Tukey correction. Kruskal–Wallis with post hoc Mann–Whitney *U* tests were used for categorical or ordinal variables (sex and *APOE* ε4 status). For modeling the trajectories of plasma p-tau epitopes as a function of Aβ burden (plasma Aβ or Aβ–PET), we corrected each plasma p-tau epitope value by age and sex. Individuals in the 15th lower percentile for Aβ–PET or the 15th highest percentile for plasma Aβ42/40 were used as anchors to *z* scores. Then, we applied a robust linear regression method (Lowess), using 1,000 robustifying iterations, with a smoother span of 0.95. The effect size of groups (Aβ^+^, Ast^+^ and Aβ^+^/Ast^+^) in relation to Aβ^−^/Ast^−^ was estimated using Cohen’s *d*, in which the dependent variable was the plasma p-tau biomarkers corrected for age and sex (Ast^+^ group included individuals positive for astrocyte reactivity but Aβ^−^, whereas the Aβ^+^ group included individuals positive for Aβ but Ast^−^). The associations between biomarkers were assessed with linear regressions accounting for age and sex. An interaction term between Aβ burden × astrocyte reactivity status/or plasma GFAP as a continuous variable was also added to each model. For analyses including all cohorts, we included cohort as a covariate to adjust for variability in differences between cohorts. For all linear regression analyses, *z* scores were centered on the mean within each cohort, and *z* scores for plasma Aβ ratio were inverted to pool plasma Aβ and Aβ–PET levels together. To determine sex effects in our findings, we included an interaction term between Aβ burden × sex on plasma p-tau considering individuals that are Ast^−^ and Ast^+^. Voxel-wise associations between biomarkers were tested using linear regressions accounting for age and sex, and adjusted for multiple comparisons using a random field theory threshold of *P* < 0.001 (ref. ^51^). We evaluated the percentage of abnormality across PET Braak-like stages by dividing the total number of voxels by the number of significant voxels after multiple comparison corrections. We measured the annual rate of progression in (^18^F)MK-6240 uptake as the difference between follow-up and baseline uptakes divided by the time between scans.

### Reporting summary

Further information on research design is available in the Nature Portfolio Reporting Summary linked to this article.

## Online content

Any methods, additional references, Nature Portfolio reporting summaries, source data, extended data, supplementary information, acknowledgements, peer review information; details of author contributions and competing interests; and statements of data and code availability are available at 10.1038/s41591-023-02380-x.

### Supplementary information

Supplementary Information Supplementary Tables 1–6 and Figs. 1 and 2.

Reporting Summary

## Figures and Tables

**Extended Data Table 1:**
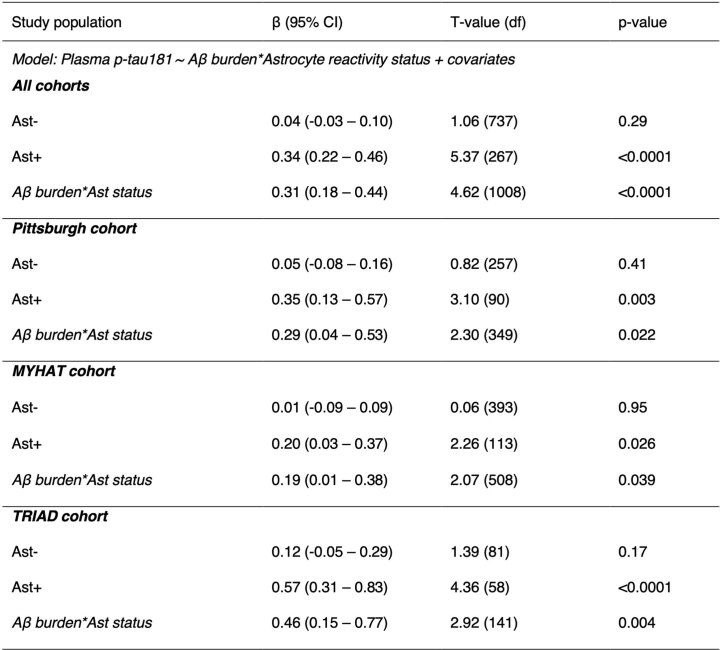
Associations between plasma p-tau181 and Aβ burden according to astrocyte reactivity status

Abbreviations: Ast−: reactive astrocyte negative; Ast+: reactive astrocyte positive; p-tau: phosphorylated tau. We inverted the values for plasma Aβ ratio in the analysis to pool plasma Aβ and Aβ-PET levels together. Covariates: age, sex, and cohort (when all studies were evaluated together). P-values were computed using linear regression models adjusted by age, sex, and cohort (when appropriate) for individuals classified as Ast− and Ast+. In addition, the Aβ burden × astrocyte reactivity status interaction was also computed.

**Extended Data Table 2:**
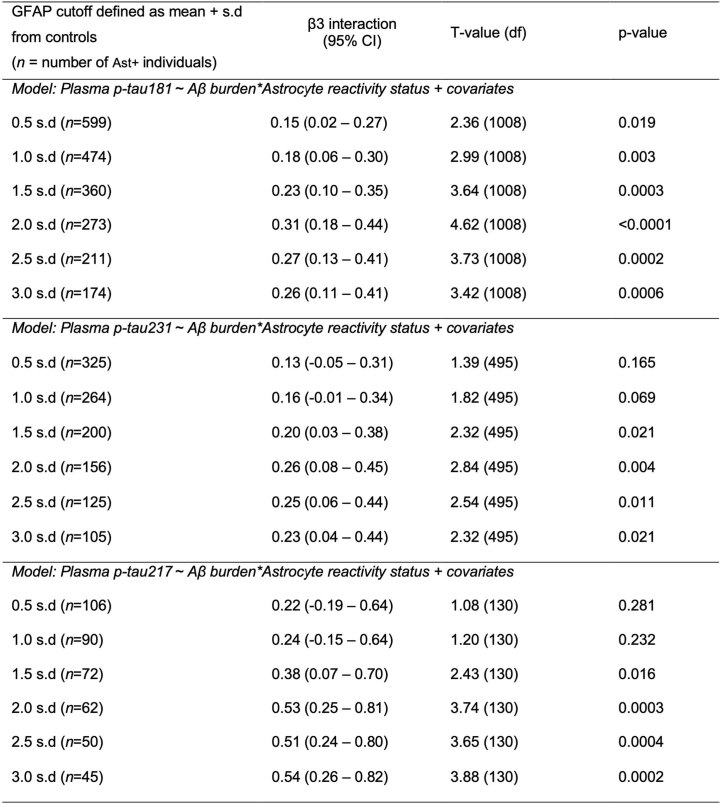
Sensitivity analyses using multiple plasma GFAP cutoffs to determine astrocyte positivity

Abbreviations: Ast−: reactive astrocyte negative; Ast+: reactive astrocyte positive; GFAP: glial fibrillary acidic protein; s.d.: standard deviation; p-tau: phosphorylated tau. The highest β-estimate was obtained using GFAP cutoff calculated as the mean + 2.0 s.d. of controls devoid of Aβ pathology for all plasma p-tau epitopes. Covariates: age, sex, and cohort. We inverted the values for plasma Aβ ratio in the analysis to pool plasma Aβ and Aβ-PET levels together. P-values were computed using linear regression models adjusted by age, sex and cohort (when appropriate) for individuals classified as Ast− and Ast+. In addition, the Aβ burden × astrocyte reactivity status interaction was also computed.

**Extended Data Table 3:**
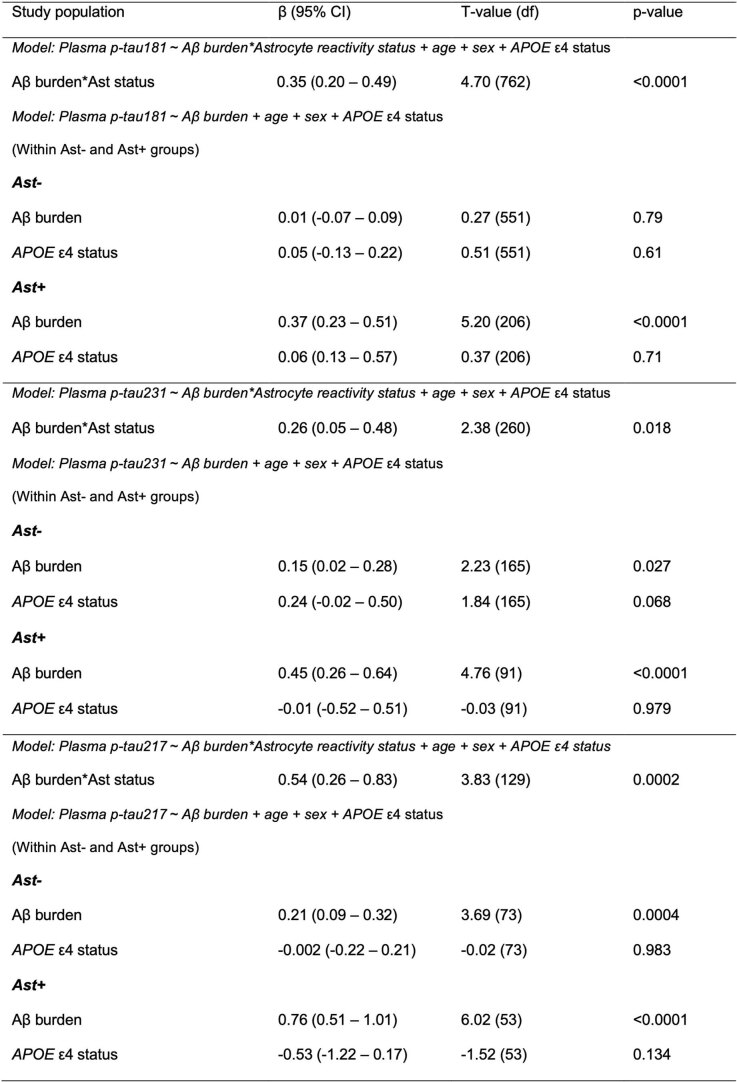
Sensitivity analysis adjusting for APOEε4 status

Abbreviations: Ast−: reactive astrocyte negative; Ast+: reactive astrocyte positive; p-tau: phosphorylated tau. We inverted the values for plasma Aβ ratio in the analysis to pool plasma Aβ and Aβ-PET levels together. P-values were computed using linear regression models adjusted by age, sex and cohort (when appropriate) for individuals classifies as Ast− and Ast+. In addition, the Aβ burden × astrocyte reactivity status interaction was also computed.

**Extended Data Table 4:**
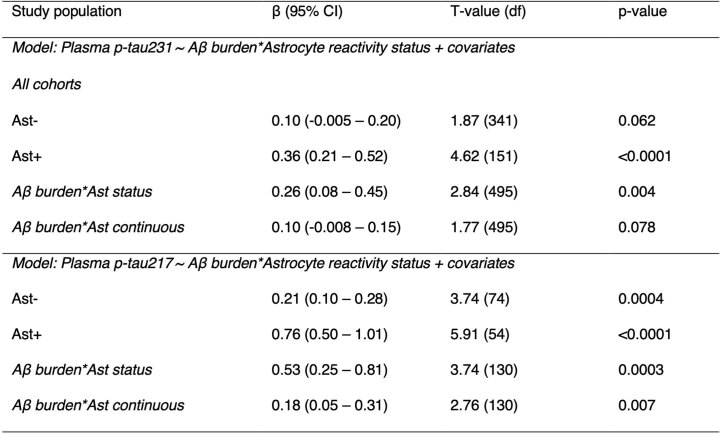
Associations between plasma p-tau231 and p-tau217 and Aβ burden according to astrocyte reactivity status

Abbreviations: Ast−: reactive astrocyte negative; Ast+: reactive astrocyte positive; p-tau: phosphorylated tau. Covariates: age, sex, and cohort. We inverted the values for plasma Aβ ratio in the analysis to pool plasma Aβ and Aβ-PET levels together. P-values were computed using linear regression models adjusted by age, sex and cohort (when appropriate) for individuals classifies as Ast− and Ast+. In addition, the Aβ burden × astrocyte reactivity status interaction was also computed.

**Extended Data Table 5:**
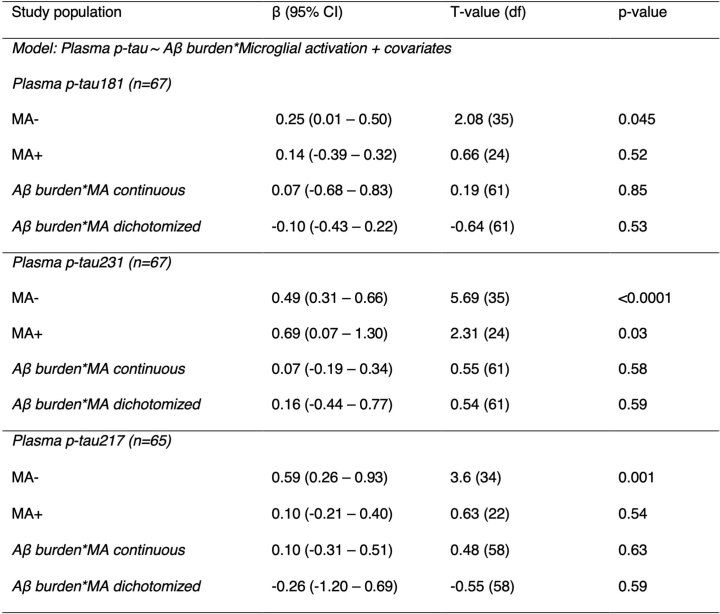
Associations between plasma p-tau and Aβ burden according to microglial activation (MA) status in the TRIAD cohort

Abbreviations: MA−: microglial activation negative; MA+: microglial activation positive; p-tau: phosphorylated tau. Covariates: age and sex. The effect of astrocyte reactivity in this subpopulation remained significant (Aβ burden × Astrocyte positivity status: *β* = 0.50, 95% CI = 0.15–0.85; t-value = 2.87; p-value = 0.006). P-values were computed using linear regression models adjusted by age, sex and cohort (when appropriate) for individuals classifies as MA− and MA+. In addition, the Aβ burden × microglial activation status interaction was also computed.

## Data Availability

All requests for raw and analyzed data and materials can be sent to the corresponding author (T.A.P.) and will be promptly reviewed by the investigators and respective institutions to verify if the request is subject to any intellectual property or confidentiality obligations. Anonymized data will be shared upon request from a qualified academic investigator for the purpose of replicating the procedures and results presented in this article. Any data and materials that can be shared will be released via a material transfer agreement. Data are not publicly available due to information that could compromise the privacy of research participants.

## Code availability

The codes used for the data analyses in our study can be requested from the corresponding author.
