# Supplementary material for: Astrocyte reactivity influences amyloid-β effects on tau pathology in preclinical Alzheimer’s disease
Source: Nat Med. 2023 May 29;29(7):1775–81. doi: 10.1038/s41591-023-02380-x (PMC10353939; doi:10.1038/s41591-023-02380-x)
Supplement: Supplementary file 1 — Supplementary Tables 1–6 and Figs. 1 and 2. [file 41591_2023_2380_MOESM1_ESM.pdf]

nature medicine

Article https://doi.org/10.1038/s41591-023-02380-x

Astrocyte reactivity influences amyloid-β effects on tau pathology in preclinical Alzheimer's disease

In the format provided by the authors and unedited

Supplementary Table 1. Demographics and key characteristics of participants of the Pittsburgh cohort.

	Aβ-/Ast- (*n* = 190)	Aβ-/Ast+ (*n* = 60)	Aβ+/Ast- (*n* = 71)	Aβ+/Ast+ (*n* = 34)
Age, mean (s.d)	63.3 (7.4)	66.9 (7.9)[a]	63.8 (7.4)[b]	69.1 (9.3)[a,c]
Sex, *n* (% female)	154 (81.1)	54 (90)	56 (78.9)	27 (79.4)
MMSE/MoCA, mean (s.d)	28.9 (1.2)/27.5 (1.8)	28.6 (1.1)/28.1 (1.7)	28.5 (1.9)/27.1 (2.1)[b]	28.8 (1.3)/27.2 (1.4)
APOEε4 (% of carriers)	13 (6.8)	1 (1.7)	13 (18.3)[b]	4 (11.8)
Education, years (s.d)	15.9 (2.2)	16.2 (2.3)	16.0 (2.5)	15.9 (3.4)
Plasma Aβ42/40, mean pg/mL (s.d)	0.083 (0.012)	0.081 (0.010)	0.058 (0.008)[a,b]	0.058 (0.007)[a,b]
Plasma GFAP, mean pg/mL (s.d)	61.2 (21.3)	132 (23.4)[a]	64.1 (20.6)[b]	137 (29.7)[a,c]
Plasma p-tau181, mean pg/mL (s.d)	15.8 (8.4)	16.5 (8.2)	15.6 (9.6)	20.9 (10.5)[a,c]
Plasma p-tau231, mean pg/mL (s.d)	13.4 (11.9)	12.7 (10.4)	11.9 (9.6)	14.9 (8.9)
Plasma NfL, mean pg/mL (s.d)	12.3 (6.4)[a]	19.9 (11.1)	12.5 (5.1)[b]	22.4 (13.5)[a,c]

Abbreviations: Aβ: Amyloid-β; A-: Aβ-negative; Ast-: reactive astrocyte negative; A+: Aβ-positive; Ast+: reactive astrocyte positive; APOEε4: Apolipoprotein ε4; MMSE: Mini-Mental State Exam; MoCA: Montreal Cognitive Assessment; GFAP: glial fibrillary acidic protein; NfL: neurofilament light chain; p-tau: phosphorylated tau; s.d: standard deviation. a = different from A-/Ast, b = different from A-/Ast+, c = different from A+/Ast-. Missing APOEε4: 133 A-/Ast-, 43 A-/Ast+, 42 A+/Ast-, 16 A+/Ast+. 91 individuals with MMSE; 264 MoCA.

Supplementary Table 2. Demographics and key characteristics of participants of the MYHAT cohort.

	Aβ-/Ast- (*n* = 294)	Aβ-/Ast+ (*n* = 67)	Aβ+/Ast- (*n* = 103)	Aβ+/Ast+ (*n* = 50)
Age, mean (s.d)	72.1 (6.2)	76.0 (7.3)[a]	71.7 (5.8)[b]	79.6 (8.7)[a,b,c]
Sex, *n* (% female)	175 (59.5)	51 (76.1)	59 (57.3)	35 (70.0)
MMSE, mean (s.d)	27.8 (3.8)	27.3 (4.1)	27.6 (3.5)	25.9 (7.6)[a]
APOEε4 (% of carriers)	55 (18.7)	16 (23.9)	19 (18.4)	15 (30)
Education, years (s.d)	14.4 (2.3)	13.7 (2.5)	14.3 (2.3)	14.2 (2.6)
Plasma Aβ42/40, mean pg/mL (s.d)	0.072 (0.006)	0.071 (0.006)	0.055 (0.007)[a,b]	0.055 (0.007)[a,b]
Plasma GFAP, mean pg/mL (s.d)	97.8 (31.4)	212 (47.9)[a]	102 (32.8)[b]	222 (52.3)[a,c]
Plasma p-tau181, mean pg/mL (s.d)	1.71 (0.84)	2.17 (0.74)[a]	1.66 (0.86)[b]	2.58 (1.15)[a,c]
Plasma NfL, mean pg/mL (s.d)	20.4 (10.2)	31.0 (15.5)[a]	22.7 (28.1)[b]	38.9 (21.9)[a,c]

Abbreviations: Aβ: Amyloid-β; Aβ-: Aβ-negative; Ast-: reactive astrocyte negative; Aβ+: Aβ-positive; Ast+: reactive astrocyte positive; APOEε4: Apolipoprotein ε4; MMSE: Mini-Mental State Exam; MoCA: Montreal Cognitive Assessment; GFAP: glial fibrillary acidic protein; NfL: neurofilament light chain; p-tau: phosphorylated tau; s.d: standard deviation. a = different from Aβ-/Ast, b = different from Aβ-/Ast+, c = different from Aβ+/Ast-. Missing APOEε4: 6 Aβ-/Ast-; 4 A+/Ast-; 1 Aβ+/Ast+.

Supplementary Table 3. Demographics and key characteristics of participants of the TRIAD cohort.

	Aβ-/Ast- (*n* = 73)	Aβ-/Ast+ (*n* = 38)	Aβ+/Ast- (*n* = 12)	Aβ+/Ast+ (*n* = 24)
Age, mean (s.d)	65.2 (11.4)	73.3 (5.2)[a]	70.4 (10.3)	72.5 (11.7)[a]
Sex, *n* (% female)	38 (52.1)	32 (84.2)[a]	7 (58.3)	17 (70.8)
MMSE, mean (s.d)	29.0 (1.05)	29.3 (0.88)	28.8 (1.19)	28.8 (1.45)
APOEε4 (% of carriers)	21 (28.8)	8 (21.1)	1 (8.3)	6 (25.0)
Education, years (s.d)	15.4 (4.0)	16.5 (3.6)	13.6 (4.0)	15.6 (3.6)
Aβ-PET, mean SUVR (s.d)	1.27 (0.1)	1.3 (0.1)	1.90 (0.27)[a,b]	1.92 (0.26)[a,b]
Plasma GFAP, mean pg/mL (s.d)	127 (33.3)	274 (69.3)[a]	141 (27.4)[b]	350 (120)[a,b,c]
Plasma p-tau181, mean pg/mL (s.d)	9.89 (4.76)	9.53 (3.28)	11.8 (3.24)	17.6 (13.3)[a,b]
Plasma p-tau231, mean pg/mL (s.d)	12.4 (5.9)	12.7 (4.5)	18.1 (5.3)	24.2 (13.4)[a,b]
Plasma p-tau217, mean pg/mL (s.d)	0.046 (0.02)	0.049 (0.02)	0.073 (0.03)	0.128 (0.12)[a,b,c]
Plasma NfL, mean pg/mL (s.d)	17.6 (8.4)	25.2 (9.4)	23.6 (11.7)	32.3 (29.4)[a]

Abbreviations: Aβ: Amyloid-β; A-: Aβ-negative; Ast-: reactive astrocyte negative; A+: Aβ-positive; Ast+: reactive astrocyte positive; APOEε4: Apolipoprotein ε4; MMSE: Mini-Mental State Exam; MoCA: Montreal Cognitive Assessment; GFAP: glial fibrillary acidic protein; NfL: neurofilament light chain; p-tau: phosphorylated tau; s.d: standard deviation; SUVR: Standardized uptake value ratio. a = different from A-/Ast-, b = different from A-/Ast+, c = different from A+/Ast-. Missing plasma p-tau217: 7 A-/Ast-, 2 A-/Ast+, 2 A+/Ast+. Missing Plasma NFl: 2 A-/Ast-; 2 A-/Ast+, 1 A+/Ast-. Missing MMSE: 1 A-/Ast-.

Supplementary Table 4. Associations between plasma NfL and Aβ burden according to astrocyte reactivity status.

Study population	β (95% CI)	T-value (df)	p-value
*Model: Plasma NfL ~ Aβ burden*Astrocyte reactivity status + covariates*			
All cohorts			
Ast-	0.03 (-0.03 – 0.09)	1.11 (736)	0.27
Ast+	0.13 (-0.02 – 0.27)	1.77 (267)	0.08
*Aβ burden*Ast status*	0.10 (-0.05 – 0.22)	1.49 (1005)	0.14
Pittsburgh cohort			
Ast-	0.02 (-0.10 – 0.10)	0.48 (257)	0.63
Ast+	0.05 (-0.31 – 0.40)	0.27 (90)	0.79

*Aβ burden*Ast status*	0.01 (-0.24 – 0.26)	0.11 (349)	0.91
MYHAT cohort			
Ast-	0.07 (-0.15 – 0.29)	0.63 (393)	0.53
Ast+	0.45 (0.01 – 0.90)	2.03 (113)	0.045
*Aβ burden*Ast status*	0.36 (-0.10 – 0.82)	1.54 (508)	0.12
TRIAD cohort			
Ast-	0.17 (0.02 – 0.31)	2.29 (78)	0.025
Ast+	0.15 (-0.13 – 0.42)	1.07 (56)	0.29
*Aβ burden*Ast status*	0.04 (-0.29 – 0.36)	0.22 (136)	0.85

Abbreviations: Ast-: reactive astrocyte negative; Ast+: reactive astrocyte positive; NfL: neurofilament light chain. Missing NfL from 5 individuals from the TRIAD cohort. Covariates: age, sex, and cohort (when all studies were evaluated together). We inverted the values for plasma Aβ ratio in the analysis to pool plasma Aβ and Aβ-PET together. P-values were computed using linear regression models adjusted by age, sex, and cohort (when appropriate) for individuals classifies as Ast- and Ast+. In addition, the Aβ burden × astrocyte reactivity status interaction was also computed.

Supplementary Table 5. Demographics and key characteristics of participants of the TRIAD cohort with available longitudinal data.

	Ast- (*n* = 38)	Ast+ (*n* = 33)	P-value
Age, mean (s.d)	65.2 (10.5)	71.6 (10.1)	0.010
Sex, *n* (% female)	19 (50)	29 (88)[a]	0.002
MMSE, mean (s.d)	29.0 (1.09)	29.2 (1.16)	0.498
APOEε4 (% of carriers)	12 (31.6)	6 (18.2)	0.307
Aβ-PET, mean SUVR (s.d)	1.34 (0.2)	1.48 (0.35)	0.048
Tau-PET temporal meta-ROI, mean SUVR (s.d)	0.86 (0.08)	0.90 (0.24)	0.411
Plasma GFAP, mean pg/mL (s.d)	130 (32.9)	288 (91.3)[a]	<0.0001
Plasma p-tau181, mean pg/mL (s.d)	9.29 (3.23)	10.3 (5.43)	0.34
Plasma p-tau231, mean pg/mL (s.d)	13.1 (6.4)	14.1 (6.2)	0.488
Plasma p-tau217, mean pg/mL (s.d)	0.049 (0.02)	0.076 (0.09)	0.089
Plasma NfL, mean pg/mL (s.d)	17.5 (8.0)	22.5 (8.4)	0.016

Abbreviations: Aβ: Amyloid-β; A-: Aβ-negative; Ast-: reactive astrocyte negative; A+: Aβ-positive; Ast+: reactive astrocyte positive; APOEε4: Apolipoprotein ε4; MMSE: Mini-Mental State Exam; GFAP: glial fibrillary acidic protein; NfL: neurofilament light chain; p-tau: phosphorylated tau; s.d: standard deviation; SUVR: Standardized uptake value ratio. Differences between groups in continuous were assessed using analysis of variance (ANOVA) with Tukey correction. Kruskal–Wallis with post-hoc Mann–Whitney U-tests were used for categorical or ordinal variables.

Supplementary Table 6. Cohort descriptions.

Cohort	Cohort description
Heart SCORE	Heart SCORE began in 2003 as a community-based participatory research study conducted in Allegheny County, PA. Initial aims of this single-site study were to improve risk stratification and evaluate mechanisms for population differences in CVD. Prespecified recruitment goals were enrollment of 2000 participants (50% black), including 800 participants at low Framingham risk, 1000 participants at intermediate or high Framingham risk, and 200 participants with established CVD. Minority recruitment was enriched through community-based initiatives in partnership with the Pittsburgh Theological Seminary and Urban League of Greater Pittsburgh and mass mailings. Participants were 45 to 75 years old at entry; individuals with a comorbid condition that was expected to limit life expectancy to <5 years or an inability to undergo annual follow-up visits were excluded. Baseline evaluation included assessment of demographics, psychosocial characteristics, and exercise and dietary habits, measurements of traditional and emerging CVD risk factors (eg, C-reactive protein, interleukin-6, lipoprotein particle sizes), evaluation for sleep disturbances, and assessments of subclinical atherosclerosis (using electron beam computed tomography scan, brachial artery ultrasonography, and finger pulse amplitude tonometry). 55 individuals from this cohort were used in our study.
HCP	This is a community-based study of brain structural and functional connectivity among cognitively normal and cognitively impaired individuals aged 50–89 years. There are currently two primary portals of entry into the study: the University of Pittsburgh Alzheimer's Disease Research Center and the Pitt + Me web portal (primarily to recruit Black individuals and Whites without college education). Additional individuals were identified through active links with the Heart SCORE, the Long Life Family Study, and by word of mouth. Each participant undergoes a brief neuropsychological test battery for group classification purposes. The test battery is based on that of the ADRC and includes the Montreal Cognitive Assessment (MoCA), verbal fluency, a 30-item visual naming test, verbal free recall, and the Rey-Osterreith Complex Figure. Classification decisions were made independently by two experienced physicians and any differences were resolved in a group discussion. We use the ADRC classification scheme for AD, MCI (both amnestic and non-amnestic), and Subjective Cognitive Complaints (SCC). 105 individuals from this cohort were used in our study.
Normal Aging Study	Elderly persons were recruited from the community for this study. Participants were recruited primarily from an ad in a local seniors' newspaper, and others were recruited from previous studies on the effect of normal aging on cognitive performance. They were not recruited from an Alzheimer research center and, therefore, had not sought treatment for dementia, nor were they recruited as the spouses or family members of patients with dementia. However, a family history of dementia was not an exclusion criterion for this study. After providing informed

	consent, participants underwent eligibility screening, including health screening and neuropsychological testing. Exclusion criteria included the presence of dementia or MCI, either amnestic or amnestic plus other areas of impairment, as well as the presence or history of other major neurological or psychiatric diseases or the use of psychoactive medications. The presence of risk factors for AD, such as being an apolipoprotein E ε4 (APOE ε4) allele carrier, was not an exclusion criterion. 32 individuals from this cohort were used in our study.
MsBrain Study	The MsBrain study aimed at understanding how symptoms relate to midlife women relate to brain health and cognitive functioning. Women were recruited from the community via advertisements, mailings, and message boards. Participants underwent physical measurements, hot flash monitoring, a blood draw, and a carotid artery ultrasound. Procedures were approved by the University of Pittsburgh Institutional Review Board. Participants provided written, informed consent. Exclusion criteria included: hysterectomy and bilateral oophorectomy; history of heart disease, stroke, arrhythmia, ovarian/gynecological cancer, pheochromocytoma, pancreatic tumor, kidney failure, seizures, Parkinson disease, Raynaud phenomenon; current pregnancy; or having used select medications in the past 3 months (oral/transdermal estrogen or progesterone, selective estrogen receptor modulators, selective serotonin reuptake inhibitors, serotonin norepinephrine reuptake inhibitors, gabapentin, insulin, β-blockers, calcium channel blockers, α-2 adrenergic agonists, and other antiarrhythmic agents). Women who had undergone uterine ablation, endarterectomy, or lymph node removal or who were undergoing dialysis or chemotherapy was also excluded. 163 individuals from this cohort were used in our study.

Supplementary Figures

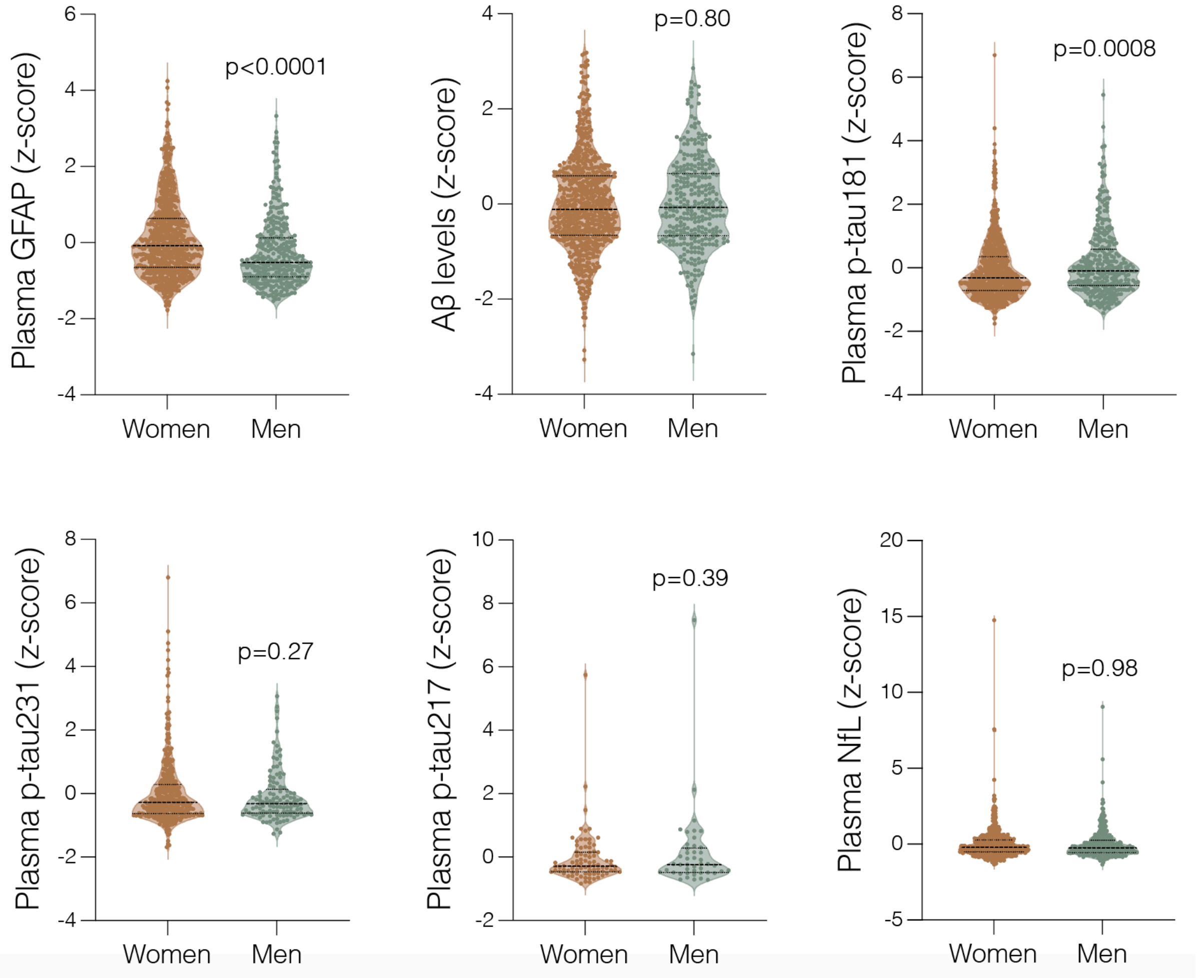

Supplementary Fig. 1. Comparison of plasma biomarker levels between men and women. **a** GFAP (n = 1,016), **b** Aβ (n = 1,016), **c** p-tau181 (n = 1,016), **d** p-tau231 (n = 502), **e** p-tau217 (n = 136) and **f** NfL (n = 1,011). Group comparisons were computed with a one-way ANCOVA adjusting for age.

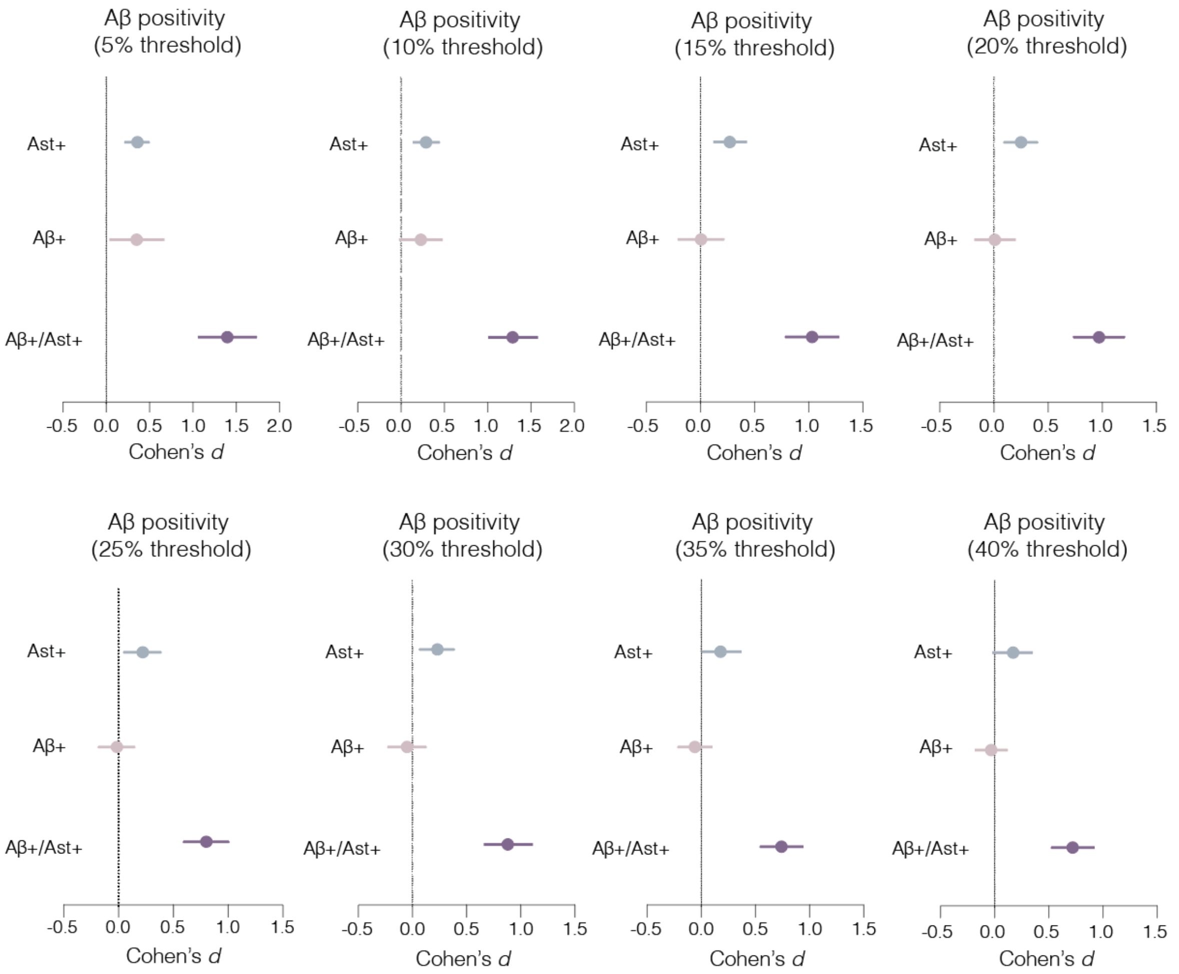

Supplementary Fig. 2. Sensitivity analysis using different cutoffs for Aβ positivity to perform the Cohen's d analysis presented in Figure 1d. We used a stepwise cutoff for Aβ positivity (from 5-40%) applied to our entire population and observed the magnitude of effect on plasma p-tau181 (accounting for age and sex) of the biomarker groups compared to the Aβ-/Ast- group (*n* = 1,016). The error bars represent the 95% CIs.
