# Supplementary material for: Astrocyte reactivity influences amyloid-β effects on tau pathology in preclinical Alzheimer’s disease
Source: Nat Med. 2023 May 29;29(7):1775–81. doi: 10.1038/s41591-023-02380-x (PMC10353939; doi:10.1038/s41591-023-02380-x)
Supplement: Supplementary file 2 — Reporting Summary [file 41591_2023_2380_MOESM2_ESM.pdf]

nature portfolio

Corresponding author(s): Tharick A. Pascoal

Last updated by author(s): Mar 14, 2023

Reporting Summary

Nature Portfolio wishes to improve the reproducibility of the work that we publish. This form provides structure for consistency and transparency in reporting. For further information on Nature Portfolio policies, see our Editorial Policies and the Editorial Policy Checklist.

Statistics

For all statistical analyses, confirm that the following items are present in the figure legend, table legend, main text, or Methods section.

n/a	Confirmed	
☐	☒	The exact sample size (*n*) for each experimental group/condition, given as a discrete number and unit of measurement
☐	☒	A statement on whether measurements were taken from distinct samples or whether the same sample was measured repeatedly
☐	☒	The statistical test(s) used AND whether they are one- or two-sided<br>*Only common tests should be described solely by name; describe more complex techniques in the Methods section.*
☐	☒	A description of all covariates tested
☐	☒	A description of any assumptions or corrections, such as tests of normality and adjustment for multiple comparisons
☐	☒	A full description of the statistical parameters including central tendency (e.g. means) or other basic estimates (e.g. regression coefficient) AND variation (e.g. standard deviation) or associated estimates of uncertainty (e.g. confidence intervals)
☐	☒	For null hypothesis testing, the test statistic (e.g. *F*, *t*, *r*) with confidence intervals, effect sizes, degrees of freedom and *P* value noted<br>*Give P values as exact values whenever suitable.*
☒	☐	For Bayesian analysis, information on the choice of priors and Markov chain Monte Carlo settings
☒	☐	For hierarchical and complex designs, identification of the appropriate level for tests and full reporting of outcomes
☐	☒	Estimates of effect sizes (e.g. Cohen's *d*, Pearson's *r*), indicating how they were calculated

Our web collection on statistics for biologists contains articles on many of the points above.

Software and code

Policy information about availability of computer code

Data collection	No software was used.
Data analysis	R statistical software version 4.2.1; MATLAB software version R2022b (9.13.0.2049777) and RMINC v1.5.3.0 (function mincLm), and VoxelStats toolbox version 1 (https://github.com/sulantha2006/VoxelStats).

For manuscripts utilizing custom algorithms or software that are central to the research but not yet described in published literature, software must be made available to editors and reviewers. We strongly encourage code deposition in a community repository (e.g. GitHub). See the Nature Portfolio guidelines for submitting code & software for further information.

Data

Policy information about availability of data

All manuscripts must include a data availability statement. This statement should provide the following information, where applicable:

- Accession codes, unique identifiers, or web links for publicly available datasets
- A description of any restrictions on data availability
- For clinical datasets or third party data, please ensure that the statement adheres to our policy

All requests for raw and analyzed data and materials can be sent to the corresponding author (TAP) and will be promptly reviewed by the investigators and respective institutions to verify if the request is subject to any intellectual property or confidentiality obligations. Anonymized data will be shared upon request from

a qualified academic investigator for the purpose of replicating the procedures and results presented in this article. Any data and materials that can be shared will be released via a material transfer agreement. Data are not publicly available due to information that could compromise the privacy of research participants.

Human research participants

Policy information about studies involving human research participants and Sex and Gender in Research.

Reporting on sex and gender	The findings presented here were observed in both men and women. However, a stronger effect of astrocyte reactivity on the association between amyloid and tau was observed in men. Sex, not gender, was used in the study design based on self-report. There is no information about disaggregated sex and gender for these individuals. Sex was added as a covariate in all models. Sex-effects were also tested in the associations between amyloid and tau.
Population characteristics	Supplemental Tables 1,2,3 and 5. Briefly, the main study population is composed by 1,016 cognitively unimpaired individuals from two research and one population-based cohort. The mean age of this population is 69.6 (std 8.9) years-old, 69.4% were women.
Recruitment	The Translational Biomarkers in Aging and Dementia (TRIAD) cohort (Montreal, Canada, https://triad.tnl-mcgill.com) comprised participants with a detailed clinical and cognitive assessment. Exclusion criteria included inability to speak English or French, inadequate visual and auditory capacities for neuropsychological assessment, active substance abuse, major surgery, recent head trauma, medical contraindication for positron emission tomography (PET) or magnetic resonance imaging (MRI), currently being enrolled in other studies, and neurological, psychiatric, or systemic comorbidities that were not adequately treated with a stable medication regimen. The Monongahela-Youghiogheny Healthy Aging Team (MYHAT) is a population-based study cohort drawn from a Rust Belt region of southwestern Pennsylvania, USA. Participants were selected by age-stratified random sampling from the publicly available voter registration lists over two time periods: 2006-2008 and 2016-2019. Inclusion criteria at study entry included: 1) 65+ years old, 2) living in a designated town, 3) not residing in long-term care settings, 4) having sufficient hearing and vision to complete neuropsychological testing, and 5) having decisional capacity. The Pittsburgh cohort is composed of research volunteers from four studies conducted at the University of Pittsburgh: the Heart Strategies Concentrating on Risk Evaluation (Heart SCORE) parent study, the Human Connectome Project (HCP), the Normal Aging study, and the MsBrain. While our cohort represents significant socioeconomic diversity, the it is mainly composed of White participants.
Ethics oversight	For TRIAD, the study was approved by the Douglas Mental Health University Institute Research Ethics Board and Montreal Neurological Institute PET working committee and all participants provided written informed consent. For MYHAT and Pittsburgh cohorts all procedures were approved by the University of Pittsburgh Institutional Review Board and all participants provided written informed consent. Participants from all cohorts received compensation commensurate with the amount of study procedures completed and duration of participation.

Note that full information on the approval of the study protocol must also be provided in the manuscript.

Field-specific reporting

Please select the one below that is the best fit for your research. If you are not sure, read the appropriate sections before making your selection.

☒ Life sciences ☐ Behavioural & social sciences ☐ Ecological, evolutionary & environmental sciences

For a reference copy of the document with all sections, see nature.com/documents/nr-reporting-summary-flat.pdf

Life sciences study design

All studies must disclose on these points even when the disclosure is negative.

Sample size	The sample size here was determined based on the number of participants with available plasma GFAP, plasma p-tau and amyloid-B (plasma or PET), from three prospective studies (MYHAT [n=514], Pittsburgh [n = 355] and TRIAD [n=147]). All participants with baseline plasma GFAP, plasma p-tau and amyloid-B data available were analyzed in this study.
Data exclusions	Individuals with no plasma GFAP, plasma p-tau and plasma or PET amyloid data were excluded from the analysis. All cognitively impaired individuals (CDR > 0 or MoCA < 25) were excluded. Young individuals (age < 25) were also excluded from the TRIAD cohort. For plasma amyloid data, outliers were removed interquartile range intervals.
Replication	Our results were replicated in three independent cohorts.
Randomization	In these cohort studies (observational studies) no allocation into experimental groups were performed, therefore randomization is not relevant to this study. Statistical analyses were controlled for potential confounding effect of age and sex.
Blinding	All biomarker (PET and plasma) collection and analyses performed in this study were performed blinded to clinical diagnosis.

Reporting for specific materials, systems and methods

We require information from authors about some types of materials, experimental systems and methods used in many studies. Here, indicate whether each material, system or method listed is relevant to your study. If you are not sure if a list item applies to your research, read the appropriate section before selecting a response.

Materials & experimental systems

n/a	Involved in the study
☒	☐ Antibodies
☒	☐ Eukaryotic cell lines
☒	☐ Palaeontology and archaeology
☒	☐ Animals and other organisms
☒	☐ Clinical data
☒	☐ Dual use research of concern

Methods

n/a	Involved in the study
☒	☐ ChIP-seq
☒	☐ Flow cytometry
☒	☐ MRI-based neuroimaging
